# Porcelain Crowns

**Published:** 1882-09

**Authors:** W. H. Shulze


### ARTICLE IV.

### *Porcelain Crowns.*

BY DR. W. H. SHULZE.

Replacing a lost tooth crown upon the remaining root has been practiced for more than a century, and yet until within a few years the practice was limited to a few methods, and these were used sparingly. In 1782 Robert Woofendale, said, "that the crown of a human tooth can be fixed to a sound root by the assistance of a screw of gold or silver." In 1814 Benjamin James said, "that some dentists fasten the new crown upon the root by driving the wire which is attached to the crown, into the canal of the root, with cotton wrapped around it to make it tight; while others place a piece of wood in the root and attach the crown to this substance." The mode preferred by Mr. James was, "simply screwing part of a gold or wood pivot into the crown and driving the other part (previously squared on its sides) into the wood socket, already perforated for its reception, and placed in the root." These were the principal methods, but owing to the difficulty and expense of obtaining sound human tooth crowns, and the unreliability of animal teeth and ivory, the practice was exceedingly limited. With the introduction of porcelain teeth the process of pivoting became more popular, and various methods were used for supplying the lost tooth crowns, until the porcelain pivot tooth, with a hole in its base to receive a wooden peg came into use. Then the principal method for many years was to attach this tooth to a pivot made of hickory wood and fitted into the enlarged root canal. But after a time the many objections to the use of wooden pivots became apparent and teeth were more frequently replaced on small gold or silver plates, secured to some of the remaining teeth, by clasps; and as cheaper bases came into use the roots were almost always extracted, and the loss supplied by the insertion of artificial plates. However, along with cheap bases came cohesive gold, and the treatment and cure of diseased roots, so that those who were suffering with abscessed teeth and disfigured with decayed crowns, could have the one restored to health and comfort, and the other to its original form, with gold. Thus for many years it was either extract the tooth or build it out with gold; indeed most of us can recall the time that we thought a tooth that could not be filled with gold had better be extracted. Of course the preservation of the natural teeth is of paramount consideration and I believe that we should bring into use every appliance, method and material which will accomplish this result. We have combated heroically against the ravages of decay among the beautiful but degenerated teeth of the past and present generation, and done much towards saving them, but how often we have been disappointed at seeing our best efforts fail; not from lack of care, thoroughness or ability, but because we had poor tooth structures to build upon and irresistible conditions of the mouth prevailed against us. With increased intelligence as to what is necessary to make good, solid tooth structure, both in regard to diet and care, we can hope that better teeth may bless the jaws of the coming men and women.

As in time the wooden peg was found defective, so we have found many defects and failures in restoring crowns with gold, and the revival of restoring the lost crown with artificial substitutes by pivoting, is gradually gaining favor again. In bringing this subject before you for consideration I should like to have had the time to have informed myself thoroughly of every method that has been brought to the notice of the profession in the last ten years, and presented them for our information and discussion, but I can only refer to a few of the many methods without any arrangement as to the priority, and add such suggestions as may be practical and beneficial.

Dr. Beers of California is accredited with the invention of gold crowns, having received a patent in 1873. The gold crowns were intended to take the place, to a great extent, of building out with gold, and in very many cases, answered a better purpose. The Richmond crown (as the gold crowns are termed) can often be used to superior advantage, and will prove serviceable when an attempt to build up with gold would prove futile. These crowns can be adapted also when the pulp is alive, the attachment being more particularly dependent upon the perfect fit around the cervical margin than a pivot in the root. But gold, from its conspicuousness in most mouths, must give way to the porcelain crown, which can be made equally useful and appear natural. During one of the periodial revivals of the old subject of the replantation of teeth, a novel plan was proposed (but whether practiced I do not know) of attaching the crown to the root. It was to extract the tooth and cut the crown off square. The porcelain tooth had a gold screw screwed into it, and was made square at the cervical base. The root canal was reamed out and the gold screw screwed into it, and then the whole replanted into the socket.

The plan of filling the root canal thoroughly with gold and attaching the crown with a gold screw, passed through a hole made for the purpose in the base of the gold, soldered to a plate tooth, was the forerunner of the Foster porcelain crown. This method is, I think, one of the best in use, but it requires much time and skill, and must necessarily be limited to the few, owing to the expense. Besides, it is adapted more particularly for incisor teeth, and cannot be used so easily for bicuspids and molars as other methods. In some cases I take the following plan, which is more applicable where good access can be had at the lingual portion of the tooth. After cutting off the crowns, take a plate tooth and fit it to the root. Fit a heavy gold plate to cover the base of the root, and solder this on the gold backing. The root canal must be enlarged with a fissure bur, having the part nearest the apex of the root slightly the largest. With a thin piece of gold make a tube that will exactly fit the hole in the root, cut it off the proper length and solder to the gold plate, which must have a hole drilled in it to correspond with the hole in the root. Now, after-sawing down the tube about half way, press it into the root canal and pack gold foil into it. The spreading of the tube against the walls of the enlarged root canal, holds the crown firmly in place. This method also involves time and skill and consequently expense, and like a great many others can be adopted only when the patient is able to compensate the dentist, so that some plan, which is less expensive, and equally practical and useful, is of course the most desirable. I think the Bonwill crown system meets this demand. For the last five years I have been gradually abandoning the laborious process of building out teeth with gold, particularly in dead incisor teeth with discolored crowns, and have found the adoption of porcelain crowns much preferable, both as to utility and appearance.

It will not be necessary for me to enter into any detailed statement or description of the best plan for replacing lost tooth crowns, but I would urge those of you who have not given this subject much consideration to turn your attention to it, for you will find that any method, almost, is easier and more satisfactory for you, and your patients who have undergone the tedious and often doubtful operation of restoration by filling, will appreciate the difference. You can often make roots, that would otherwise be extracted, useful and obviate the necessity of wearing an artificial plate.

The past year I have used the Bonwill crowns in most all cases, for I find this method simple and practical. Ina number of cases I have prepared the root and attached the crown at one sitting, with less labor and fatigue to myself and patient than would be required in filling an ordinary cavity with gold in a position difficult of access. It is preferable, however, to secure the pin in the root and let the amalgam become hardened before attaching the crown. I will not prolong this article by describing the method which you are more or less familar with, but refer you to Dr. Bonwill's articles in the "*Cosmos.*" However, I will cite a couple of cases: One that I had last March, and more than a year ago, which will illustrate the appreciation of the patient. A gentleman who resided in New York city for many years (living in our prohibition city now) called on me to attend to his teeth. He had many fine gold fillings in his teeth, and as he expressed it, "You see doctor I have been through the mill." The incisors were filled on the labial surface, and on the approximal sides. The point of the left central had broken off, carrying with it the approximal and labial fillings, and left the half of the lingual wall standing with the root filling. He wanted the tooth built down. I proposed attaching a crown, and after a little persuasion, for he had heard only of wooden pivot teeth, he consented. I had difficulty in getting a match for the color of his teeth, but I finally succeeded, after having three different teeth made at Philadelphia. I had a hole made in the labial surface, which I filled with gold to correspond with the right central, and when the crown was attached it required a mouth mirror to discover which was the artificial substitute. He was pleased with the result, expressed his gratification at being relieved from the long filling operation, has several times assured me of the stability and comfort of "his new tooth," as well as his surprise that the "thing could be done." The other case was for a lady, for whom, ten years ago, Dr. Callahan spent six hours in building up a gold crown upon frail walls on a lower molar. The length of time the filling stood is a compliment to the gentleman's ability, for it was one of her main grinders. She came into my office in distress with the filling in her pocket-book. I told her I was glad the tooth had broken away, for she was always worrying about losing that tooth, but she wanted it built up again. I proposed a much easier method, and at once cut off the remaining portion of the crown, and took an impression of the space, and adjoining and antagonizing teeth. As the space was quite large, I sent the plaster cast to Philadelphia to have a special crown made. When received I set the two pins in the roots and attached the crown in an hour. It is firm and with the exception of the small amalgam fillings in the crown, it would be taken for a perfect natural tooth upon examination. The lady has expressed her thanks often, and told me that she would rather pay twice as much for an artificial crown and be spared the tedious operation of filling.—*Proceedings of Kansas State Dental Association.*

